# Biology

**Published:** 1875-01-15

**Authors:** N. S. Davis


### MEDICAL EXAMINER.

A

*Semi-Monthly Journal of Medical Sciences.*

EDITED BY N. S. DAVIS, M.D., AND F. H. DAVIS, M.D.

No. II. CHICAGO, JAN. 15, 1875. VOL. XVI.

### Original Communications.

### BIOLOGY.

A PAPER READ TO THE EVANSTON PHILOSOPHICAL SOCIETY, DEC. 29TH, 1874, BY N. S. DAVIS, A. M., M. D., ETC.

THE word *Biology*, was first introduced into common use by Treviranus at the commencement of the present century.

It means literally, a discourse on life; and is now very generally used to designate that branch of science relating to the origin, development and phenomena of living matter.

By some it is used as synonymous with physiology.

It would be better, however, to restrict the word to its original application, as indicating an inquiry into the origin and essential properties of matter capable of manifesting the phenomena of life.

Through all the past ages of literature and philosophy, few topics have been more fruitful in eliciting discussions, observations and experiments, developing a great variety of facts and theories, and yet without a satisfactory adjustment of the latter to a full explanation of the former, than the one under consideration.

In a paper recently read before this Society, it was stated, substantially, that the universe consists of *matter*, *force*, and mind or spirit; the two former essentially passive, the latter alone capable of spontaneous or self-action. But strictly speaking, is there any such thing as *force*, except as a condition or property of either matter or mind? Aside from the operations of mind, can we find any proof of the existence of *force* except as a property or condition of matter? To speak of *force* as one of the distinct entities of the universe, parallel with matter and mind, but distinct from both the latter, appears to me erroneous. Neither can I conceive of such a thing or idea as *passive force.* The ideas properly conveyed by the two words are antagonistic, and therefore not capable of correct application to the same thing at the same time.

Yet we not only meet at every turn with expressions clearly conveying the idea of *force* as a separate entity or subject of thought, but also representing it as a fixed quantity in the universe, appearing in different forms or modes of action, but never annihilated. For instance, a ton of coal is said to contain a certain amount of *force*, meaning thereby, that under certain circumstances in connection with oxygen, it would be capable of developing a certain amount of that peculiar *mode of motion* in matter termed in common language, *heat.*

As a figurative expression it would be generally understood correctly; but is it scientifically correct? Has the ton of coal in reality any force except its specific gravity and the cohesion of its atoms? That it has the capacity, when its temperature is properly elevated, to enter rapidly into combination with oxygen, and by such union, so to increase the movements in the atoms of matter as to constitute a given amount of force, is true.

But the *capacity* to develop force under certain conditions, is not itself force. As well might we say an egg is a hen, or the germinal cell of the ovum, a man. It is proper to say, that a ton of coal and a certain quantity of oxygen are capable, by their union, of developing a certain amount of force.

The *force* itself exists only while the union is taking place.

The capacity to develop the force in some other form may still remain, because neither the oxygen nor the carbon is annihilated by their union. As the so-called cosmic forces of the material world, heat, light, electricity, attraction, etc., are simply exhibitions of material properties and modes of motion, and as no atom of matter is annihilated by simply changing its relation to other atoms, it is proper to say, that the *capacity* for evolving these forces in connection with the material universe is, in the aggregate, always the same. But while the capacity to evolve force is the same, the amount actually being evolved varies with each passing hour. My object in presenting these preliminary remarks, is to invite attention to the necessity, especially in scientific and philosophical discussions, of more exactness in our modes of expression, that we may avoid vagueness and confusion of thought, and the conveyance of imperfect or erroneous ideas. And in no field of inquiry is this caution more necessary than in that pertaining to the study of living matter. For here, more than elsewhere, the word *force* has been used in the most vague and latitudinous manner.

The ancients had their vis vitae, vis anima, vis generatrix, vis medicatrix, vis conservatrix naturæ, etc., which they used on all needful occasions to explain whatever to them was otherwise unexplainable. So also at the present time, writers assume the existence of a vital, biotic or life *force;* a neurotic or nerve force; a myotic or muscular force; a reparative or conservative force, etc., and invest these respective forces with whatever attributes or powers are necessary to serve their purposes. If we interpret the language applied to them literally, we find them not only invested with individuality and intelligent action, but also with many of the qualities of matter, such as quantity and motion. For instance, it is common to hear certain articles of food and drink spoken of as capable of conversion into life force or nerve force; to hear these forces spoken of as capable of increase, accumulation and exhaustion.

Again, the vis conservatrix or reparative force, in medical literature especially, is invested with the most remarkable attributes. The expressions used, not only imply a separate and independent existence, but also the highest order of intelligent action; a veritable goddess enthroned in the living body and capable of acting in every direction to guard the citadel of life. It has seemed to me that the assumption of the existence of forces not capable of proof, and the investment of them with just such attributes and capabilities as our fancy might deem necessary, was not only unphilosophical, but greatly detrimental to the progress of true science. That there are specific and absolute differences that distinguish living from dead matter, is too obvious to need illustration. Both, however, require to be studied in the same manner, and with an equally careful adherence to observed facts and inductive laws. If we take up a piece of inorganic matter for investigation, we analyze it to find the kind of matter of which it is made; and we note its shape, density, and particular arrangement of its atoms, to determine the properties by which they are held together. If we pursue the same course in regard to living matter we shall arrive at results equally satisfactory. That there are certain properties peculiar to, and inherent in all organic matter, so long as it retains the capacity to exhibit the phenomena of life, is evident from facts familiar to us all. Take for example the simplest form of organization, the germinal cell of the ovum, or the chit or germinal part of the seed. Each is destitute of all trace of either capillary vessel or nerve, yet each is susceptible to the impression of certain exterior agents or influences, and whenever these are applied, a series of regular and determinate changes commence, constituting the active phenomena of life. It requires but a moment of careful logical thought to recognize here the existence of two inherent elementary properties. One imparts to the cell or mass of bioplasm the capacity to receive impressions, and hence I have called it *susceptibility*. The other causes the atomic changes that result from the impressions received, to follow certain laws, both in the addition of new atoms and the liberation of old ones; and I have, therefore, called it *vital affinity*. *Susceptibility* and *vital affinity* are the elementary properties of all organized living matter. It is the possession of these properties that gives to the protoplasm of Mr. Huxley, and the bioplasm of Mr. Beale, all their peculiarities and capabilities of development.

It would be a waste of time to speculate as to the nature of these properties. They constitute the peculiar and elementary forces of the organic world, and can be recognized and studied only by their effects, in the same manner that we recognize and study heat, electricity, attraction, as elementary forces of the inorganic world. You suspend two inorganic substances in water, and if they unite, forming a new material, you say the union was the result of a property in the combining bodies which you call chemical affinity. You do not see the property or force, yet for that reason you do not doubt its existence. So if we place the germinal cell of the animal or vegetable in certain relations we find its atoms uniting with other atoms of matter, and forming not a new and homogeneous compound, as in the display of chemical affinity, but a complex and progressive series of additions constituting growth or development; and we call the property in the germinal cell by which these changes are effected, *vital affinity*. These properties, susceptibility and vital affinity, are elementary and inherent in all organized living atoms of matter, whether vegetable or animal. Deprive the germ of either or both of these properties and it immediately becomes subject to the same laws that govern inorganic matter. Expose it to warmth and moisture, ever so sedulously, and instead of the phenomena of life, you have only those of disintegration and decay.

It has been claimed by some, that these properties of living matter are simply correllations of those controlling inorganic or dead matter. But they are so radically different, both in origin and modes of action, as to defeat all attempts to trace their identity with either heat, electricity, or any of the varieties of attraction. For instance, chemical affinity causes the atoms of matter to unite in certain definite proportions, and the union thus formed to remain stationary; while vital affinity, once called into activity, continues its influence over the atomic or molecular changes through periods of time more or less definite, inducing combinations and decompositions in successive order until a certain result is reached. Again, the properties of inorganic matter are universal in their presence, being capable of detection in all forms of matter, organic and inorganic, while susceptibility and vital affinity are manifested only in matter the molecules of which have assumed a definite arrangement under the influence of some prior organic body.

In other words, there are no known circumstances under which the molecules of inorganic or of organic matter *spontaneously* unite in such a manner as to exhibit the properties of living matter. I am aware that Pouchet and others claim to have demonstrated by experiments, that some of the infusoria, as vibriones and bacteria, are capable of originating spontaneously in solutions containing organic matter from which all prior germs or spores had been excluded; but other equally careful experimenters have arrived at opposite conclusions. And it only requires a glance at the progress of the science of life during the last two centuries to be satisfied that there is no such phenomenon in nature as spontaneous generation.

In the days of Aristotle, many fishes, reptiles, worms, and all insects were thought to originate spontaneously. Hardly had this error been corrected in regard to these several species, by the discovery of their ova, when the numerous varieties of Entozoa began to attract attention as animals springing, de novo, from the simple aggregation of organic molecules. The appearance of maggots in meat, larva in cheese, and worms in the alimentary canal, were very generally regarded as specimens of spontaneous generation, until the experiments of Redi, in 1668, demonstrated the facts to be otherwise. It was then thought that the axiom of Harvey, "Omne Animal ex Ovo," was of universal application. But only a few years later, 1675, the discovery of animalcules or infusoria in water and other fluids, by Lieuwenhoek, opened a new and extensive field for observation; and at a later period gave rise to a renewal of the discussions concerning spontaneous generation. The invention of microscopes and their application to the study of these innumerable species of minute objects in air, earth, and water, have enabled us already to trace the propagation of many of them by the evolution of ova, spores or germs, and others by segmentation and budding, leaving at the present day only a very few of the most minute living organisms undetermined in their mode of propagation. Even Pouchet claimed to find in his solutions the spontaneous appearance of only two or three varieties, namely, the vibriones, bacteria and nonads.

If it is true, however, that during the last two centuries the progress of exact or experimental science has driven the advocates of spontaneous generation from worms and insects, down to two or three of the most minute varieties of the infusoria, we are fully justified in assuming, that further progress by the same methods will rob them of even those.

The Harvenian axiom, that all animals spring from eggs, and all vegetables from seeds, is not strictly correct. For we find some of the more minute and simple organizations of both animal and vegetable nature, to propagate their species by simple division or segmentation, instead of ova.

Without dwelling further on the modes of propagation, we simply assume the position that all organized living matter is possessed of the two properties heretofore named, susceptibility and vital affinity, and that these properties, together with the special aggregation of molecules in which they inhere, are in all instances derived from a prior living parent. Hence, both the special molecular arrangements and the specific character of the properties governing them, must be identical with those of the parent from which they were derived. And as all the molecular movements constituting nutrition, growth, and metamorphosis or disintegration in living matter, take place under the direction of these properties, it follows as a logical necessity that every bud, segment, or ovum, will follow in its development through all stages from the simple germinal cell to the most complex animal, the special type of the parent from which it was derived. If the parent was a simple aggregation of bioplasm, constituting a nonad or vibrio, with neither recognizable vessels or nerves, the propagating segment will develop into the same simple structure and nothing more.

If the parent is a vertebrate with vessels, nerves, muscles, and all the complex organs of the higher orders of animals, the germinal cell of the propagating ovum will contain molecules or atoms of bioplasm, representing each typical structure of the mature animal, and under the guidance of these elementary properties, will develop in the same order and reach the same complex maturity. So exactly do the properties of the germ represent those of the parent, that when the latter have been materially altered by disease, the former partake of the same modification and thus transmit the disease hereditarily from one generation to another. If these propositions are true, it follows that the Darwinian idea of progressive development from a lower to a higher order of living beings, by the gradual change of one species, order, or genus of plants or animals into another, is contrary to a fundamental physiological law of living matter. That the elementary properties of which we have been speaking, are capable of being modified in their operation by the influence of exterior agencies, mental and physical, is true. But such modification is limited to simple increase or diminution of activity, or to defective supply of material, and consequently shows itself, not in a change of the type of organization, but only in the degree of perfection attained in the processes of growth.

The so-called law of the "survival of the fittest" or strongest, may apply to the individual members of each species, but cannot be extended in such a manner as to transform the "fittest" of one species into the most unfit or "weakest" of another species. There is no plainer or more persistent law recognizable in the material world, than that like begets like, through all grades of living objects. Not only does this physiological law, founded on the origin and nature of the properties controlling all the molecular changes in living matter, negative the idea of progressive development of one species into another; but the radically different anatomical plans on which the different general divisions of the animal kingdom are based, renders the development of one into another, a physical impossibility. For instance, the more perfect the growth or development of an animal constructed on the plan of a radiate, the further will it become removed from one constructed on the plan of a mollusk, an articulate, or a vertebrate Neither can any possible perfection in the growth of an animal constructed on the anatomical plan of an articulate, bring it into proximity to the construction of a vertebrate.

The same thing is shown in the radical differences in the modes of development in the ova or germs of each division of the animal kingdom.

If, as we have intimated, every aggregation of molecules constituting a vegetable or animal germ, capable of manifesting the phenomena of life, is derived from a living parent, and possessed of such elementary properties as will guide each successive molecular change in a direction to make the resulting plant or animal of the same type as the parent, it must be inferred that every such germ contains at least one molecule typical of each primary structure found in the mature plant or animal.

It might be thought that this would necessitate a large number of typical molecules in the germ, especially of the higher orders of animals. A closer study, however, will show that only a few really typical or elementary structures are required for the complete development of the most complex of all animals, man. All vegetables and the lowest species of animals, present but a single structure, consisting of cells variously arranged, and exhibiting no other functions than those of assimilation or growth and elimination or excretion. As we ascend in the scale of being, we find added successively, elastic fibrous tissue, capillary vascular, nervous, and muscular tissues. Thus, all the organized living parts of the human body, are resolvable into five primary structures or forms of organization, namely, the cellular or secretory; the elastic fibrous; the capillary vascular; the muscular, and the nervous. And each of these has its own specific function or office to perform. The first imbibes from the materials in contact with it, certain substances, causes their union into new forms either for its own growth or for elimination as a secretion, or for both. The second, simply forms a tough, elastic bond of union, and support for all the other structures. The third consists of capillary tubes adapted to the movement of fluids through them, and to free exosmose and endosmose through their walls. The special office of the fourth is contraction, by which it becomes the active motor force of animal bodies. The fifth possesses the double function of receiving and transmitting impressions. Hence the germ of even the most complex animal, need contain but five typical molecules to represent all the primary structures of living matter. Each of these typical molecules being endowed with the properties, susceptibility and vital affinity, would be capable of responding to the presence of exterior matter, selecting from it the materials for its own development, and rejecting all the rest. But each must, from an absolute law of necessity, follow its own special type. Were this not so, we would constantly find the different structures of the body mixed in inextricable confusion. And yet this necessity for following the typical law of molecular growth presents another anatomical barrier to the theory of evolution or progressive development of one species into another. Through the whole range of anatomical, physiological, and pathological investigations, I am not aware of the discovery of a single instance in which a tissue of lower type of organization has been transformed into one of a higher type. For instance, we never find elastic fibres transformed into capillary vessels; the latter into muscular fibres; or muscular fibres into nerve structure. Whatever apparent transformations of tissue have been observed, have invariably been in the opposite direction, namely, from the higher to the lower form. Having already occupied quite as much time as the Society has allotted to the essayists on these occasions, I will close by stating, that the foregoing observations relate to the elementary properties of all living matter, and to the primary function of each distinct structure, as they are manifested in the various grades of vegetable and animal bodies, and without any reference to the purely mental part of man. The latter, so far as its connection with the material organization can be traced, has its special seat in a portion of the nervous structure, through which alone its operations are made known to us.

